# Does timing of neoadjuvant chemotherapy influence the prognosis in patients with early triple negative breast cancer?

**DOI:** 10.1007/s00432-023-05060-y

**Published:** 2023-07-07

**Authors:** Maria Eleni Hatzipanagiotou, Miriam Pigerl, Michael Gerken, Sophie Räpple, Verena Zeltner, Madeleine Hetterich, Peter Ugocsai, Miriam Fernandez-Pacheco, Elisabeth Christine Inwald, Monika Klinkhammer-Schalke, Olaf Ortmann, Stephan Seitz

**Affiliations:** 1 Department of Gynecology and Obstetrics, University Medical Centre Regensburg, Landshuter Straße 65, 93053 Regensburg, Germany; 2 Tumor Center Regensburg - Centre for Quality Management and Health Services Research, University of Regensburg, Regensburg, Germany; 3 Bavarian Cancer Registry, Regional Centre Regensburg, Bavarian Health and Food Safety Authority, Regensburg, Germany; 4 Department of Gynecology and Obstetrics, Maria Eleni Hatzipanagiotou, University Medical Centre Regensburg, Landshuter Straße 65, 93053 Regensburg, Germany

**Keywords:** Time to systemic therapy, Triple negative breast cancer, Neoadjuvant chemotherapy, Clinical cancer registry data

## Abstract

**Purpose:**

For patients with triple negative breast cancer (TNBC), the optimal time to initiate neoadjuvant chemotherapy (TTNC) is unknown. This study evaluates the association between TTNC and survival in patients with early TNBC.

**Methods:**

A retrospective study using data from of a cohort of TNBC patients diagnosed between January 1, 2010 to December 31, 2018 registered in the Tumor Centre Regensburg was performed. Data included demographics, pathology, treatment, recurrence, and survival. Interval to treatment was defined as days from pathology diagnosis of TNBC to first dose of neoadjuvant chemotherapy (NACT). The Kaplan–Meier and Cox regression methods were used to evaluate the impact of TTNC on overall survival (OS) and 5 year OS.

**Results:**

A total of 270 patients were included. Median follow up was 3.5 years. The 5-year OS estimates according to TTNC were 77.4%, 66.9%, 82.3%, 80.6%, 88.3%, 58.3%, 71.1% and 66.7% in patients who received NACT within 0–14, 15–21, 22–28, 29–35, 36–42, 43–49, 50–56 and > 56 days after diagnosis. Patients who received systemic therapy early had the highest estimated mean OS of 8.4 years, while patients who received systemic therapy after more than 56 days survived an estimated 3.3 years.

**Conclusion:**

The optimal time interval between diagnosis and NACT remains to be determined. However, starting NACT more than 42 days after diagnosis of TNBC seems to reduce survival. Therefore, it is strongly recommended to carry out the treatment in a certified breast center with appropriate structures, in order to enable an adequate and timely care.

## Introduction

About 15–20% of breast cancers do not express estrogen (ER) or progesterone receptors (PgR) (≤ 1%) or show human epidermal growth factor receptor 2 (HER 2) overexpression or amplification (Sporikova et al. 2018). These triple negative breast cancers (TNBCs) usually have an aggressive tumor biology associated with a young age at diagnosis of less than 40 years (Hudis and Gianni 2011). Most TNBCs not only metastasize early in the course of the disease, but tend to develop prognostically unfavorable visceral and central nervous system metastases (Lin et al. 2008). In comparison with other subtypes of breast cancer of the same stage, the survival rates of patients with TNBC are worse. The mortality rate of TNBC is 40% within the first 5 years after diagnosis (Dent et al. 2007). Therapeutic options for patients with TNBC have been limited, but in the last years new therapeutic options are arising from the rapidly increasing knowledge on the pathogenesis and tumor biology (Schneeweiss et al. 2019; Wesolowski et al. 2022; Prat et al. 2013; Schmid et al. 2020). Due to its specific molecular phenotype, TNBC is not sensitive to endocrine therapy or molecular targeted therapy. Thus chemotherapy in combination with immunotherapy depending on tumor stage is currently standard of systemic therapy in early TNBC (Wesolowski et al. 2022; Chaudhary et al. 2018). Several randomized clinical trials have demonstrated that adjuvant and neoadjuvant administration of the same chemotherapy regimen yields similar results in disease-free survival (DFS) and overall survival (OS) (Early Breast Cancer Trialists’ Collaborative Group 2018). Neoadjuvant chemotherapy (NACT) is the preferred treatment approach for early TNBC (Chaudhary et al. 2018; Burstein et al. 2019; Pusztai et al. 2019). The use of NACT leads to better operability with a higher rate of breast-conserving therapy and results in clinical and pathological downstaging of involved axillary lymph nodes (Schneeweiss et al. 2019; Pusztai et al. 2019). In addition to these clinical benefits, the use of NACT provides the opportunity to obtain early information about the responsiveness of the primary tumor to chemotherapy, so that individualized therapeutical strategies can be administered (Tutt et al. 2021; Masuda et al. 2017). Clinical and pathologic responses provide important prognostic information (Minckwitz et al. 2012). Pathologic complete remission (pCR; = ypT0/is ypN0) after NACT is associated with a longer event-free survival and overall survival (Schneeweiss et al. 2019; Liedtke et al. 2008). The current standard of care for NACT in women with TNBC is a combination of anthracyclines and taxanes (Schneeweiss et al. 2019; Korde et al. 2021). The addition of carboplatin increases the rates of pCR and improves DFS (Korde et al. 2021; Minckwitz et al. 2014; Saleh et al. 2021). Recent clinical trials allowed to develop promising immunotherapeutic strategies and put immunotherapy in combination with NACT as a new standard of care in TNBC (Schmid et al. 2020; Korde et al. 2022). In patients with TNBC, pCR rates of up to 65% can be achieved (Schmid et al. 2020). In patients who do respond with pCR postneoadjuvant therapy leads to prolonged DFS and OS (Tutt et al. 2021; Masuda et al. 2017). If neoadjuvant or adjuvant chemotherapy is administered for breast cancer, the duration should be 18–24 weeks (Krebsgesellschaft and Krebshilfe 2021). The effect of time to adjuvant chemotherapy administration (TTAC) in all breast cancer subtypes has been evaluated in several studies with different results (Chavez-MacGregor et al. 2016; Eastman et al. 2013; Pomponio et al. 2019; Gagliato et al. 2014; Li et al. 2018). In contrast there is very limited evidence on the impact of time to initiation of neoadjuvant chemotherapy (TTNC) on patient outcomes (Livingston-Rosanoff et al. 2020; Melo Gagliato et al. 2020). Both studies evaluating time to TTAC and studies evaluating TTNC show contradictory results (Chavez-MacGregor et al. 2016; Eastman et al. 2013; Pomponio et al. 2019; Gagliato et al. 2014; Li et al. 2018; Livingston-Rosanoff et al. 2020; Melo Gagliato et al. 2020). Published data suggest that a potential temporal impact may be particularly important in triple-negative breast cancer, due to the aggressive tumor biology (Chavez-MacGregor et al. 2016; Gagliato et al. 2014). To clarify this important clinical problem, we evaluated whether TTNC is associated with survival in patients with early TNBC in a large population-based study using the Tumor Centre Regensburg clinical cancer registry database. In addition, we evaluated the determinants of delayed chemotherapy administration.

## Methods

### Study population and variables

**Fig. 1:**
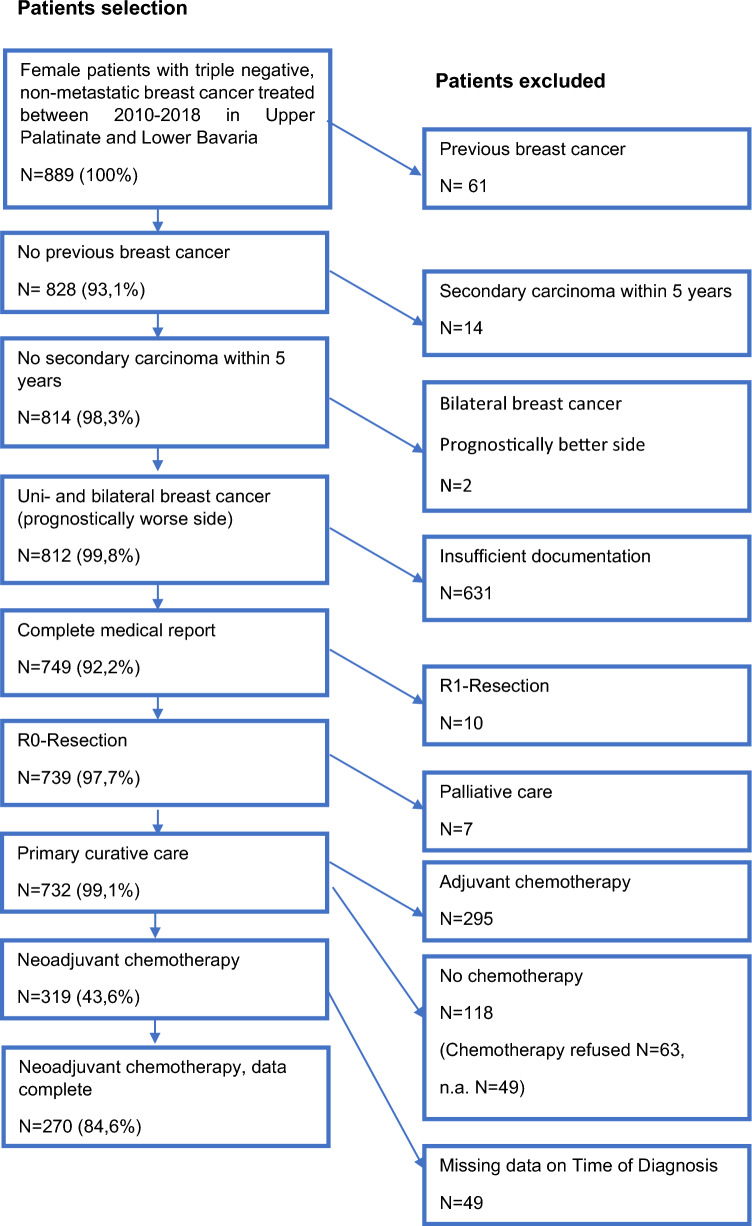
Depiction of the study collective

In this retrospective cohort study, clinical cancer registry data from the Tumor Centre Regensburg from patients with TNBC with a focus on diagnosis, treatment and progression were used for evaluation. A population of more than 2.2 million people including Upper Palatinate and Lower Bavaria is covered in this population-based regional cancer registry. Electronic sheets of documentation contain information about diagnosis, course of disease, therapies, and the complete follow-up of patients. These population‐based data originate from medical reports, pathology reports and follow‐up records. Diagnosis and treatment modalities, course of disease and several histologic parameters are documented as well as long-term follow-up including locoregional or distant recurrence and mortality. The Tumor Center Regensburg has documented tumor diseases in the Upper Palatinate and Lower Bavaria since 1991 and is integrated into the Centre for Quality Assurance and Health Services Research at the University of Regensburg. The population consisted of women living in Upper Palatinate and Lower Bavaria diagnosed and treated with NACT for TNBC and recorded by the Tumor Center Regensburg in the period from January 1, 2010 to December 31, 2018. Figure 1 describes inclusion and exclusion for the final study collective. Data analysis was performed between September 2021 and July 2022.

Patient information, including demographic characteristics and variables related to cancer diagnosis and treatment, were abstracted from medical records by tumor registries as part of routine procedures. We analyzed the following parameters: Tumor site location, lymphatic vessel invasion, vein invasion, stage, grading, Ki67, ER, PR, and Her2neu status, date of diagnosis of the primary tumor, age, last date of follow up, date of recurrence date, date of death, Charlson comorbidity index, date of primary surgery and date of first application of chemotherapy. Regarding cancer stage, the clinical stage at diagnosis was determined by clinical evaluation, in addition to breast and axillary imaging.

### Statistical analysis

Patients were categorized according to the time between TNBC pathologic diagnosis and the application of the first dose of neoadjuvant chemotherapy (in days) into eight subgroups: ≤ 14, 15–21, 22–28, 29–35, 36–42, 43–49, 50–56, > 56 days. This interval was defined as TTNC. The exact start for TTNC was the day of the pathologic diagnosis. The end date was the first day of neoadjuvant chemotherapy. Descriptive statistics were used to evaluate the characteristics of the patient population according to TTNC. Follow-up was calculated using the reverse Kaplan–Meier method. Survival time was calculated in days from the date of breast cancer diagnosis to the date of last follow-up, the date of death, or the cut-off date. Patients who were known to be alive at the study cutoff date of August 31, 2021, were censored on that date. The Tumor Center Regensburg regularly updates vital patient status information and active hospital follow-ups through linkages with state and national health offices and queries to the residents' registration offices. Recurrence-free survival was determined as the difference between the date of diagnosis of the primary tumor to the last living date, recurrence date, date of death or cut-off date. For cumulative recurrence rates, the date of diagnosis and the respective recurrence date (local, locoregional, or distant metastasis) were used, censored by the last living date, cut-off date, or date of death. Univariable analyses of cumulative overall survival, cumulative recurrence rates, and recurrence-free survival were implemented using the Kaplan–Meier method, and the log-rank test was used to compare differences between groups. Mean survival time in years and a 5-years survival rate was analyzed. Median survival was not reached by the cut-off date. Using a multivariable Cox proportional hazard model, we examined the impact of TTNC as a categorical variable on OS. Variables in the multivariable model were selected a priori and included age at diagnosis, Charlson comorbidity index, year of diagnosis, site location, lymphatic vessel invasion, venous invasion, stage, grading, Ki67, and type of primary surgery. Additionally a supplementary backward stepwise selection was applied. Data collection and statistical analysis were performed using IBM SPSS Statistic 25 (IBM Corporation, Armonk, NY). Hazard ratios (HR), p-values, and 95% confidence intervals (CI) were calculated for each model. All tests were 2-sided, and *p *< 0.05 was considered significant and *p* < 0.001 was considered highly significant.

## Results

We identified 37,382 patients with malignant neoplasms of the mammary gland that were coded by the „International Statistical Classification of Diseases and Related Health Problems” (ICD-10) Code C50. After exclusion as shown in Fig. 1 the final study cohort included 732 patients, of which 319 patients (43.6%) received NACT for TNBC, and 295 patients (40.3%) received adjuvant chemotherapy for TNBC. No chemotherapy was administered to 118 patients (16.1%), 63 of them refused any type of chemotherapy (8.6%) and in 49 patients (6.7%) the reason for renouncing chemotherapy was not documented, in 6 cases documentation on chemotherapy application was missing. 49 patients were excluded because of missing data on the exact time of diagnosis, so the TTNC could not be evaluated. Here we report the results of the study subgroup of 270 patients with TNBC treated with NACT and valid TTNC. Among the 270 patients included in the final analysis the mean age at diagnosis was 53.2 years. 146 women (54.1%) were postmenopausal and 124 (45.9%) were premenopausal at the time of diagnosis of TNBC. The median TTNC was 28.9 days. A total of 34 (12.6%) patients started chemotherapy within 14 days after diagnosis; 61 (22.6%) between 15–21 days; 63 (23.3%) between 22–28 days, 39 (14.4%) between 29–35 days, 37 (13.7%) between 36–42 days, 19 (7.0%) between 43–49 days, 9 (3.3%) between 50–56 days, and 8 (3.0%) started chemotherapy 56 or more days after diagnosis. 234 (86.7%) patients started NACT within 42 days after diagnosis of TNBC, 262 (97.0%) started within 56 days after diagnosis. Patient, tumor, and treatment characteristics stratified by TTNC are shown in Table 1.

**Table 1:** Patient, tumor, and treatment characteristics stratified by TTNC

	Days from histological confirmation of diagnosis to start of neoadjuvant systemic therapy																	
	< = 14		15–21		22–28		29–35		36–42		43–49		50–56		> 56		Total	
	*N*	*n* %	*N*	*n* %	*N*	*n* %	*N*	*n* %	*N*	*n* %	*N*	*n* %	*N*	*n* %	*N*	*n* %	*N*	*n* %
Age at diagnosis																		
< 40	8	23.5	12	19.7	6	9.5	4	10.3	5	13.5	4	21.1	1	11.1	1	12.5	41	15.2
< 50	13	38.2	18	29.5	14	22.2	10	25.6	8	21.6	1	5.3	2	22.2	1	12.5	67	24.8
50–59	9	26.5	21	34.4	21	33.3	13	33.3	16	43.2	3	15.8	4	44.4	3	37.5	90	33.3
60–69	2	5.9	9	14.8	14	22.2	6	15.4	5	13.5	9	47.4	2	22.2	2	25.0	49	18.1
≥ 70	2	5.9	1	1.6	8	12.7	6	15.4	3	8.1	2	10.5	0	0.0	1	12.5	23	8.5
Menopausal status																		
Prem	22	64.7	32	52.5	26	41.3	16	41.0	15	40.5	6	31.6	5	55.6	2	25.0	124	45.9
Postm	12	35.3	29	47.5	37	58.7	23	59.0	22	59.5	13	68.4	4	44.4	6	75.0	146	54.1
Co-morbidities																		
No	28	82.4	51	83.6	52	82.5	31	79.5	26	70.3	11	57.9	4	44.4	3	37.5	206	76.3
Yes	4	11.8	7	11.5	6	9.5	4	10.3	8	21.6	6	31.6	3	33.3	5	62.5	43	15.9
n.a	2	5.9	3	4.9	5	7.9	4	10.3	3	8.1	2	10.5	2	22.2	0	0.0	21	7.8
Stage																		
IA/B	3	8.8	13	21.3	17	27.0	8	20.5	7	18.9	6	31.6	5	55.6	2	25.0	61	22.6
IIA	14	41.2	21	34.4	24	38.1	19	48.7	21	56.8	7	36.8	3	33.3	2	25.0	111	41.1
IIB	11	32.4	18	29.5	12	19.0	9	23.1	8	21.6	3	15.8	0	0.0	3	37.5	64	23.7
III	5	14.7	9	14.8	9	14.3	3	7.7	1	2.7	3	15.8	1	11.1	1	12.5	32	11.9
n.a	1	2.9	0	0.0	1	1.6	0	0.0	0	0.0	0	0.0	0	0.0	0	0.0	2	0.7
Tumor size																		
T1	7	20.6	18	29.5	18	28.6	10	25.6	11	29.7	8	42.1	7	77.8	2	25.0	81	30.0
T2	22	64.7	33	54.1	34	54.0	27	69.2	26	70.3	7	36.8	1	11.1	4	50.0	154	57.0
T3	3	8.8	9	14.8	6	9.5	1	2.6	0	0.0	2	10.5	1	11.1	2	25.0	24	8.9
T4	1	2.9	1	1.6	4	6.3	1	2.6	0	0.0	2	10.5	0	0.0	0	0.0	9	3.3
n.a	1	2.9	0	0.0	1	1.6	0	0.0	0	0.0	0	0.0	0	0.0	0	0.0	2	0.7
Nodal status																		
N0	16	47.1	32	52.5	45	71.4	25	64.1	25	67.6	13	68.4	6	66.7	5	62.5	167	61.9
N1	16	47.1	24	39.3	15	23.8	13	33.3	11	29.7	6	31.6	3	33.3	3	37.5	91	33.7
N2	1	2.9	4	6.6	1	1.6	0	0.0	1	2.7	0	0.0	0	0.0	0	0.0	7	2.6
N3	1	2.9	1	1.6	2	3.2	1	2.6	0	0.0	0	0.0	0	0.0	0	0.0	5	1.9
Grading																		
G1/2	3	8.8	7	11.5	7	11.1	5	12.8	6	16.2	1	5.3	0	0.0	0	0.0	29	10.7
G3/4	24	70.6	43	70.5	45	71.4	21	53.8	15	40.5	9	47.4	7	77.8	4	50.0	168	62.2
n.a	7	20.6	11	18.0	11	17.5	13	33.3	16	43.2	9	47.4	2	22.2	4	50.0	73	27.0
Lymphatic vessel invasion																		
L0	21	61.8	44	72.1	52	82.5	31	79.5	33	89.2	14	73.7	8	88.9	7	87.5	210	77.8
L1	5	14.7	10	16.4	6	9.5	2	5.1	1	2.7	4	21.1	0	0.0	1	12.5	29	10.7
n.a	8	23.5	7	11.5	5	7.9	6	15.4	3	8.1	1	5.3	1	11.1	0	0.0	31	11.5
Vein invasion																		
V0	23	67.6	51	83.6	57	90.5	31	79.5	34	91.9	16	84.2	8	88.9	8	100.0	228	84.4
V1	2	5.9	2	3.3	1	1.6	2	5.1	0	0.0	1	5.3	0	0.0	0	0.0	8	3.0
n.a	9	26.5	8	13.1	5	7.9	6	15.4	3	8.1	2	10.5	1	11.1	0	0.0	34	12.6
Ki67																		
0–25	6	17.6	13	21.3	16	25.4	7	17.9	11	29.7	4	21.1	1	11.1	1	12.5	59	21.9
> 25	28	82.4	47	77.0	47	74.6	30	76.9	25	67.6	15	78.9	8	88.9	7	87.5	207	76.7
n.a	0	0.0	1	1.6	0	0.0	2	5.1	1	2.7	0	0.0	0	0.0	0	0.0	4	1.5
Type of surgery																		
BCT	26	76.5	56	91.8	56	88.9	34	87.2	34	91.9	11	57.9	7	77.8	5	62.5	229	84.8
Mastectomy	7	20.6	5	8.2	7	11.1	5	12.8	3	8.1	8	42.1	1	11.1	3	37.5	39	14.4
n.a	1	2.9	0	0.0	0	0.0	0	0.0	0	0.0	0	0.0	1	11.1	0	0.0	2	0.7
Total	34	100.0	61	100.0	63	100.0	39	100.0	37	100.0	19	100.0	9	100.0	8	100.0	270	100.0

*n.a.* not available

Median follow up was 3.5 years. At the time of analysis 48 patients (17.8%) had died. Overall, patients had an estimated mean OS of 7.6 years after diagnosis. Those who received systemic therapy early had the highest estimated OS of 8.4 years, while patients who received systemic therapy after more than 56 days survived an estimated 3.3 years. From the cut-off value of 42 days after diagnosis there was a trend towards worse survival. with only 4.7 years OS when starting NACT after 43 days, 4.1 years when starting NACT after 50 days and 3.3 years when starting NACT after 56 days or more (Table 2).

**Table 2:** Estimated mean OS times and 5-year-OS rates according to TTNC

TTNC (days)	No. Patients *n*	Mean OS (years)	Standard-deviation	Mean (95% CI)	5-year OS rate (%)
≤ 14	34	8.37	0.82	(6.76–9.98)	77.4
15–21	61	6.24	0.48	(5.30–7.18)	66.9
22–28	63	6.68	0.42	(5.80–7.51)	82.3
29–35	39	5.52	0.31	(4.90–6.13)	80.6
36–42	37	7.03	0.39	(6.26–7.80)	88.3
43–49	19	4.72	0.45	(3.84–5.61)	58.3
50–56	9	4.10	0.50	(3.12–5.07)	71.1
> 56	8	3.30	0.46	(2.38–4.21)	66.7
All	270	7.56	0.43	(6.73–8.39)	

**Fig. 2:**
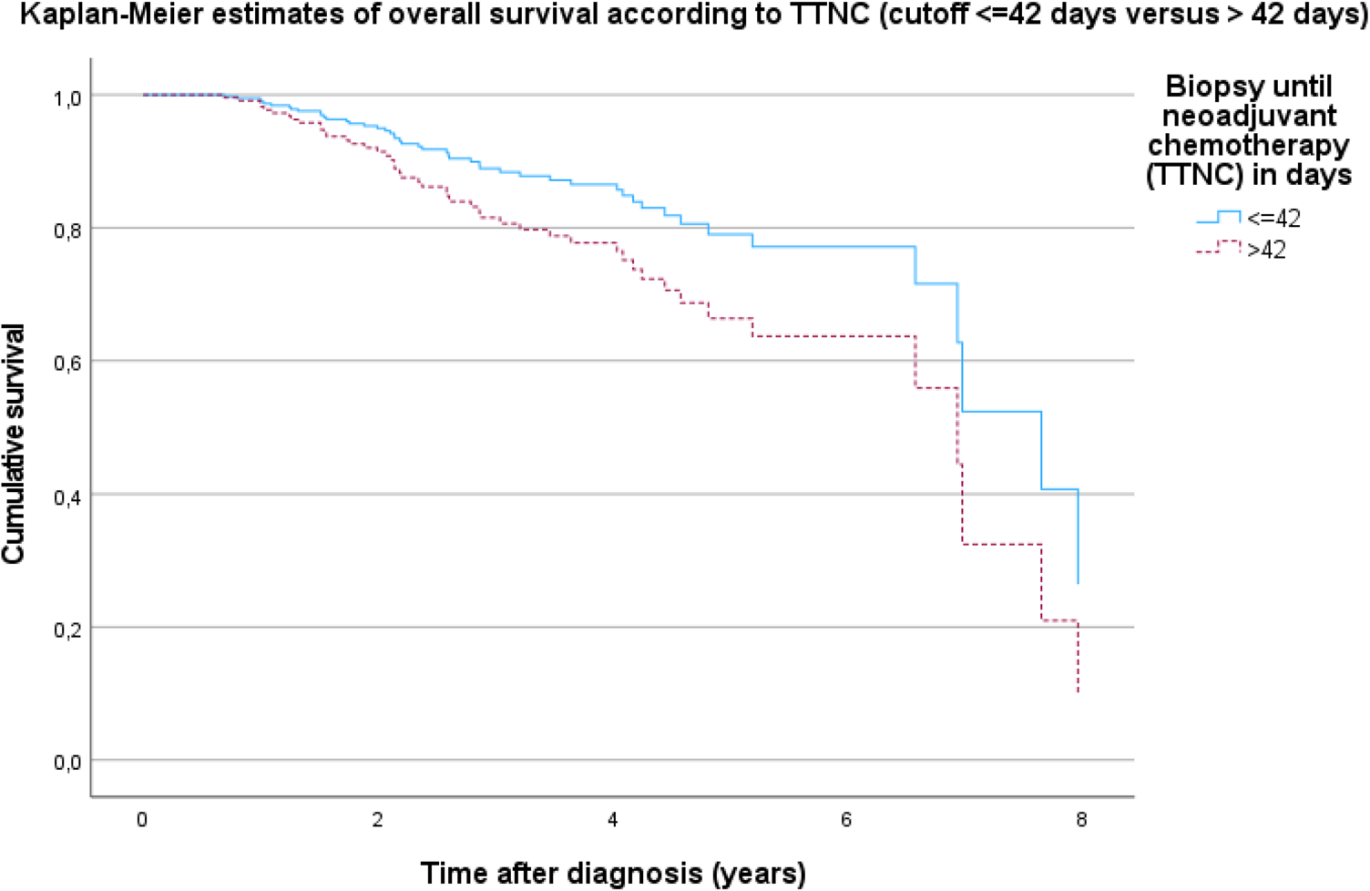
Kaplan–Meier estimates of overall survival according to TTNC (cutoff ≤ 42 days versus > 42 days), *p* = 0.484

The analysis demonstrated a trend for better 5-years OS with earlier initiation of systemic therapy. If systemic therapy is started after 56 or more days, the 5-years OS rate is only 66.7% (Table 2). The pairwise comparisons of the groups with the log rank test (Mantel-Cox) showed that patients who already received systemic neoadjuvant chemotherapy within the first 2 weeks after the diagnosis are more likely to survive than those who receive systemic therapy after more than 56 days (*p* = 0.054, data not shown). Although this is barely not significant, there is a trend towards worse survival in patients who received NACT after 56 days. The best 5-year OS was seen in patients who received system therapy within 42 days, 234 patients (86.6%) had been treated within 42 days after diagnosis, 36 patients (13.3%) did not meet this time criterion. Figure 2 illustrates Kaplan–Meier cumulative survival rates for TTNC ≤ 42 days versus TTNC > 42 days.

**Fig. 3:**
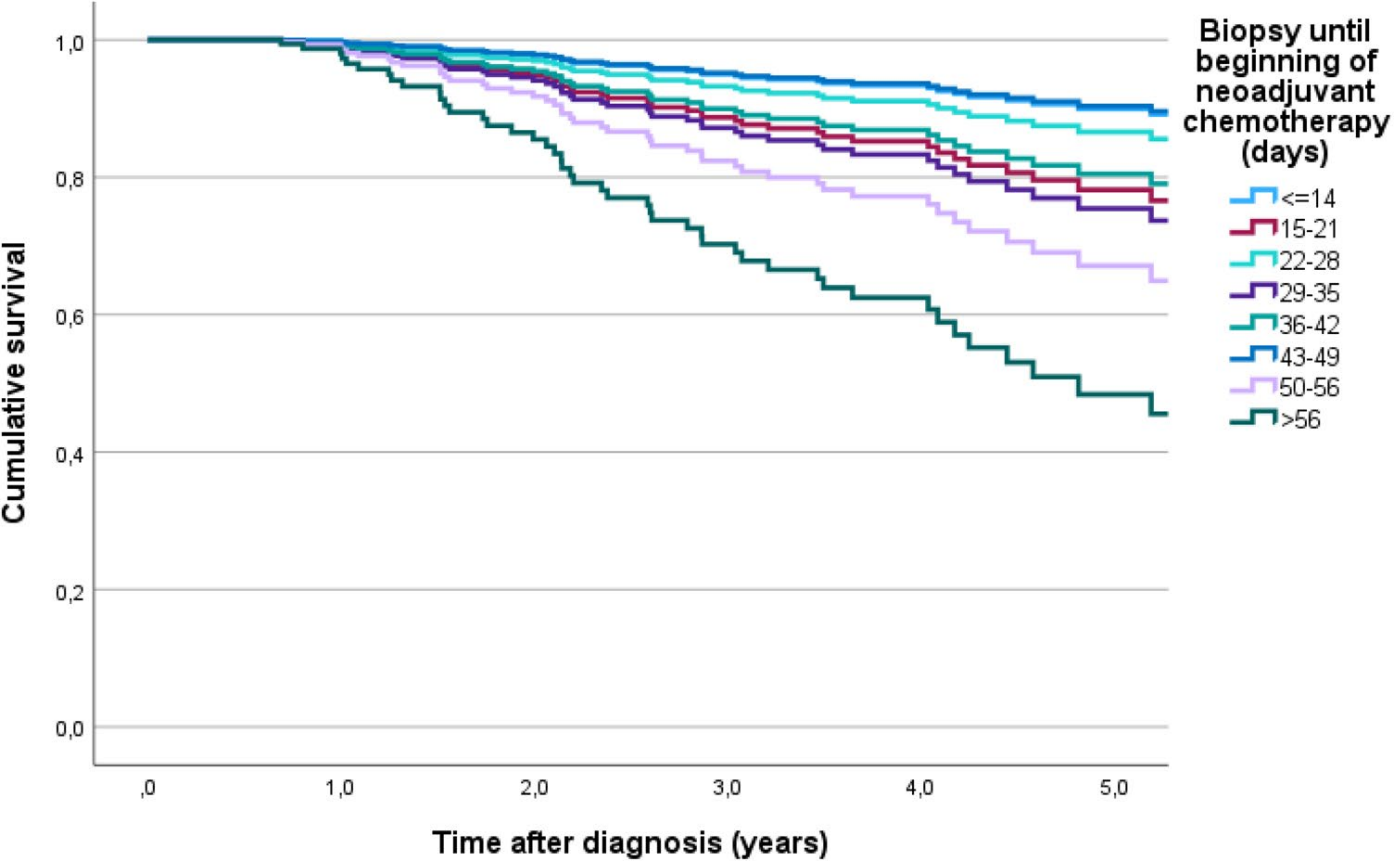
Kaplan–Meier estimates of 5-year overall survival according to TTNC

Figure 3 shows the Kaplan–Meier estimates of 5-year survival according to TTNC in each time interval. It illustrates the decrease in 5-year survival with increasing delay in the initiation of NACT. The groups with TTNC of 50–56 days or > 56 days experience the worse OS. Although statistical significance is not reached, the curves clearly demonstrate the tendency of worse OS with increasing TTNC. In the pairwise comparisons of temporal groupings using the log-rank test (Mantel-Cox), it was observed that patients who received systemic neoadjuvant chemotherapy within the first two weeks after histological confirmation of diagnosis by biopsy had better survival than patients who received systemic therapy after more than 56 days (*p* = 0.054). Patients who received systemic therapy within 3 weeks had better survival compared to those who received it after 4 weeks (*p* = 0.095) and compared to those who received it after 6 weeks (*p* = 0.090). In the group of patients who received systemic therapy after 4 weeks. better survival was observed compared to patients who received it after more than 56 days (*p* = 0.069).

In the multivariable Cox-regression analyses for OS we observed that factors associated with worse OS included lymphatic vessel invasion, mastectomy and grading (data not shown). A supplementary backward stepwise selection showed a significant hazard ratio of 4.675 (95% CI 1.050–20.815; *p* = 0.043) for patients with a TTNC of more than 56 days compared to patients with TTNC of less than 14 days. Here the significant prognostic variables were grading, lymphatic vessel invasion and type of surgery.

## Discussion

Our study focused on the analysis of survival according to TTNC in patients with TNBC under routine clinical conditions. We could show a clear trend towards reduced survival if TTNC exceeded more than 42 days. The magnitude of the adverse effect of delayed administration of NACT appeared to be even greater when administration was delayed by more than 56 days. The results indicate a clinically meaningful, albeit not statistically significant association between treatment delay and worse OS. The association between treatment delay and decreased OS seems clear but statistical significance was not reached presumably due to small numbers in the different subgroups of TTNC. Regarding timing of adjuvant chemotherapy in patients TNBC we could observe statistically significant reduced OS when adjuvant chemotherapy was delayed for more than 42 days after surgery (submitted). In the catchment area of the Tumor Center Regensburg, 86.7% of the patients were treated within a period of 42 days. Our study suggests that patients with lymphatic vessel invasion and higher grading experience worse OS. Regarding factors influencing TTNC, it must be considered that after histological confirmation an appointment for adequate counseling, further staging examinations, counseling regarding hereditary breast cancer with, eventually, genetic counseling and molecular genetic testing must be performed. Upon diagnosis of TNBC in younger women, referral for fertility preservation should also take place before treatment starts. The complex care and the aggressive tumor biology of TNBC underline the relevance of this study, which aims to explore the optimal time interval between diagnosis and initiation of therapy. In previous studies it has become evident that delays in adjuvant chemotherapy initiation are associated with worse DFS and OS among patients with early stage breast cancer (Chavez-MacGregor et al. 2016; Gagliato et al. 2014; Li et al. 2018; Biagi et al. 2011). Regarding time to adjuvant chemotherapy Chavez-MacGregor et al. showed that delayed adjuvant chemotherapy was particularly detrimental among patients with triple-negative breast cancer (Chavez-MacGregor et al. 2016). State of the art treatment of TNBC includes the application of NACT. Given the aggressive biology of TNBC and high risk of distant recurrence (Hudis and Gianni 2011; Lin et al. 2008; Dent et al. 2007), it is of particular interest to define appropriate time intervals from diagnosis to initiation of NACT to prevent an avoidable deterioration of the prognosis due to the exceeding of critical time intervals. Timing of NACT remains an area of controversy. Various studies investigated the impact of time to surgery after the completion of NACT (Sanford et al. 2016; Omarini et al. 2017; Sutton et al. 2020), whereas there is little data from research on timing of NACT with contradictory results (Livingston-Rosanoff et al. 2020; Melo Gagliato et al. 2020; Loibl et al. 2017). Loibl et al. evaluated in an explorative analysis the relationship between the time interval from diagnosis to NACT and the time from last chemotherapy administration to breast definitive surgery and outcomes. They included 9127 participants from neoadjuvant clinical trials and did not find an influence of delays in time to NACT on DFS or OS. The TTNC cutoffs evaluated were ≤ 4 vs > 4 weeks. likely explaining the difference in results when compared with our study, in which a clear tendency to deleterious impact in OS was seen among patients who initiated NACT 42 days (= 6 weeks) or more after diagnosis. The median TTNC was 23 days, compared to 28.9 days in our study. Loibl et al. concluded that time interval of starting NACT might be uncritical (Loibl et al. 2017). our study data does not support this assumption. A further retrospective analysis (*n* = 12,806) by Livingston-Rosanoff et al. evaluated whether delays in NACT initiation would impact patient survival in women with stage I-III breast cancer utilizing the National Cancer Database of the United States. They neither found an association between delays in NACT initiation and patient survival for Her2 + and TNBC. Their definition of delayed NACT was after 3 weeks for triple negative disease, also in contrast to the cutoff values of 42 and 56 days in our study. possibly explaining the difference in results. In addition, the median follow up was limited with 35 months (range 2–61) for TNBC and the authors point out that longer follow-up may reveal a difference in survival attributable to TTNC (Livingston-Rosanoff et al. 2020). In contrast to these studies De Melo Gagliatoa el al. observed in a large retrospective cohort (*N* = 5137) that in patients with stage I or II disease a delay in NACT initiation of 31–61 days and ≥ 61 days after diagnosis was associated with an increased risk of death, Interestingly, consistent with our findings, the HR for TNBC suggested an association between treatment delay and increased risk but statistical significance was not reached in the group of TNBC presumably due to small numbers (Melo Gagliato et al. 2020). Livingston et al. hypothesize that the introduction of systemic therapy earlier in the treatment timeline, before surgery, may mitigate the impact of any delays that could subsequently develop, which may explain the different findings regarding delays in starting NACT versus delays in starting adjuvant chemotherapy (Livingston-Rosanoff et al. 2020). Although significance was not reached in our study the results still indicate, that in primary non-metastatic triple-negative breast cancer, there are critical time intervals from the date of diagnosis to the start of NACT.

A limitation of our population-based study is the retrospective nature of the study which involves possible confounders. Our cohort is a large cohort of TNBC and the majority of patients was treated in accordance with national and international guidelines under routine conditions in a certified breast cancer center. We also reduced the confounders with the inclusion of important clinical factors (age at diagnosis, concomitant disease, site location, stage, grading, lymphatic vessel invasion, venous invasion, Ki-67, nodal status) in the multivariable analysis, however, the possibility of additional confounders cannot be excluded. It is possible that due to the small number of patients in the TTNC category of 42–55 days (*N* = 28) and > 56 days (*N* = 8) statistical significance could not be reached. Median follow up in the present study was 42.4 months. Longer follow-up might have lead to significant differences in OS when NACT was administered later than 42 days after diagnosis. We are not able to evaluate the effect of delays beyond 56 days, since 86.9% of the patients started NACT within the critical interval of 6 weeks after diagnosis. One advantage of the study is that data of all patients in the catchment area of the Tumor Center Regensburg were documented over several years in a population-based, comprehensive manner and not in a clinical trial setting. Thus, the present study can reliably reflect routine oncological care and outcome quality. It is shown that treatment of patients with TNBC in certified breast cancer centers leads to a start of NACT after diagnosis within 42 days in the vast majority of patients. The results of this work suggest that patients with TNBC and delayed initiation of NACT have poorer OS. The optimal time to start NACT remains a topic of fundamental clinical importance. Prospective data would be ideal to finally answer this important question but clinical trials addressing this issue will likely not be undertaken since they will be considered unethical.

It may be concluded from this large population-based study on patients with primary non-metastatic TNBC receiving NACT that there are critical time intervals from the date of diagnosis to the start of NACT, with reduced OS when NACT was applied later than 42 days after diagnosis. Consultation, therapy planning and implementation in patients with TNBC are complex. Multidisciplinary teams in certified breast centers with appropriate structures should focus on coordination of care and timely treatment planning and delivery.

## Data Availability

The datasets generated during and analyzed during the current study are not publicly available due to maintenance and privacy of tumor registry data but are available from the corresponding author on reasonable request.
